# A fluid-structure interaction model accounting arterial vessels as a key part of the blood-flow engine for the analysis of cardiovascular diseases

**DOI:** 10.3389/fbioe.2022.981187

**Published:** 2022-08-19

**Authors:** Heming Cheng, Gen Li, Jifeng Dai, Ke Zhang, Tianrui Xu, Liuchuang Wei, Xue Zhang, Dongfang Ding, Jie Hou, Jianyun Li, Jiangping Zhuang, Kaijun Tan, Ran Guo

**Affiliations:** ^1^ Department of Mechanics, Kunming University of Science and Technology, Kunming, China; ^2^ Faculty of Civil Engineering and Mechanics, Kunming University of Science and Technology, Kunming, China; ^3^ Department of Hydraulic Engineering, Kunming University of Science and Technology, Kunming, China; ^4^ Faculty of Life Science and Technology, Kunming University of Science and Technology, Kunming, China

**Keywords:** cardio-cerebrovascular diseases, strain energy, arterial vessels, physiological parameters, blood supply, fluid-structure interaction

## Abstract

According to the classical Windkessel model, the heart is the only power source for blood flow, while the arterial system is assumed to be an elastic chamber that acts as a channel and buffer for blood circulation. In this paper we show that in addition to the power provided by the heart for blood circulation, strain energy stored in deformed arterial vessels *in vivo* can be transformed into mechanical work to propel blood flow. A quantitative relationship between the strain energy increment and functional (systolic, diastolic, mean and pulse blood pressure) and structural (stiffness, diameter and wall thickness) parameters of the aorta is described. In addition, details of blood flow across the aorta remain unclear due to changes in functional and other physiological parameters. Based on the arterial strain energy and fluid-structure interaction theory, the relationship between physiological parameters and blood supply to organs was studied, and a corresponding mathematical model was developed. The findings provided a new understanding about blood-flow circulation, that is, cardiac output allows blood to enter the aorta at an initial rate, and then strain energy stored in the elastic arteries pushes blood toward distal organs and tissues. Organ blood supply is a key factor in cardio-cerebrovascular diseases (CCVD), which are caused by changes in blood supply in combination with multiple physiological parameters. Also, some physiological parameters are affected by changes in blood supply, and vice versa. The model can explain the pathophysiological mechanisms of chronic diseases such as CCVD and hypertension among others, and the results are in good agreement with epidemiological studies of CCVD.

## 1 Introduction

Cardio-cerebrovascular diseases (CCVD) are among the most common diseases causing death and disability in humans. They kill 15 million people worldwide each year and impose a large medical and economic burden on society. CCVD refers to ischemic or hemorrhagic diseases of the brain, heart, body tissues and other organs caused by hyperlipidemia, high blood viscosity, atherosclerosis, hypertension, high uric acid content and other factors, and are characterized by high morbidity, disability and mortality. The pathogenesis of CCVD is characterized by many physiological phenomena such as blood pressure, obesity, sympathetic nervous system changes, vascular stiffness, high blood lipid content and blood viscosity, as well as poor habits such as a sedentary lifestyle, lack of physical exercise, poor sleep habits, high fat and high sodium diets, smoking, and mental anxiety, among other factors.

According to the classic Windkessel model, the heart is the only power source for blood flow, while the arterial system is assumed to be an elastic chamber that only acts as a channel and buffer for blood circulation. Studies have shown that the heart of a healthy person at rest pumps 5–6 L/min of blood, and the amount of blood needed is 7.6–9.2 $\times 10^{3}Kg$ per day, in other words, cardiac output power at rest is about 0.9–1.9 W (Wang et al., 2020). Average daily cardiac output typically falls with age from 9.91 (age 23.6 years) to 5.91 (age 82) $\times 10^{3}Kg$ (Brandfonbrener et al., 1955; Idema et al., 1994), with a corresponding reduction in cardiac output power from 1.44 to 0.86 W. It is hard to imagine that blood can be pumped through the entire body by such low power. We believe that there are other sources of energy in the body for powering blood flow, including that from the elastic arterial system *in vivo*. Active elastic arteries store strain energy, which can be converted into mechanical work to propel blood flow.

Various mechanical properties of arteries have been studied from different aspects since Fung (Fung 2013). Biomechanical parameters of intact or separated arterial tissues (intima, media and adventitia) such as arterial stiffness, compliance, microstructure of elastic and collagen fibers, smooth muscle cells located within the artery wall, have been identified using methods such as planar or inflation tests to serve as a reference for clinical cardiovascular disease (CVD) diagnosis, treatment and monitoring (Niestrawska et al., 2016; Hoffman et al., 2017; Amabili et al., 2019; Ramachandra and Humphrey 2019; Lisický et al., 2021a). Also, constitutive models with different levels of complexity and number of parameters also have been developed, such as phenomenological extensions of Fung’s model (Fung et al., 1979) and structure-based versions of the GOH (Gasser-Ogden-Holzhapfel) model (Gasser et al., 2006; Rachev and Shazly 2019; Lisický et al., 2021b; Holzapfel et al., 2021), to describe and predict the mechanical behaviors of arterial tissues. Meanwhile, changes in histological and mechanical properties of pathological arterial tissues with intima damage, atherosclerosis, aneurysm, and calcification could serve as a first-hand reference for clinical studies and practices (Desyatova et al., 2017; Zareh et al., 2019; Noble et al., 2020; Thrivikraman et al., 2020; Lisický et al., 2021b). However, elastic blood vessels *in vivo* are deformable tubes that are pre-stretched. Little attention has been paid to the storage of strain energy in these elastic vessels.

A large number of studies have been carried out on risk factors for CCVD incidence and mortality as a function of a single physiological variable, and useful results have been achieved in statistical clinical studies. The effects of structural features of arterial vessels on hypertension and chronic CVD have previously been studied. Aortic stiffness is closely associated with hypertensive diseases and is an independent predictor of fatal stroke in patients with essential hypertension. Arterial stiffness can be assessed noninvasively by measurement of pulse wave velocity, which is a simple and reproducible method (Laurent et al., 2001a, b; Boutouyrie et al., 2002; Laurent et al., 2003; Wu et al., 2012; Ecobici and Stoicescu 2017; Lyle and Raaz. 2017; Virdis 2018; Tomiyama 2020). The association of blood pressure (BP) with risk factors for CVD morbidity and mortality has been explored. BP is a powerful cardiovascular (CV) risk factor that acts on the arterial wall and is responsible in part for various CV events, such as cerebrovascular incidents and ischemic heart diseases (Safar 2008; Verdecchia et al., 2012; Wu et al., 2012; Malyszko et al., 2013). The effects of blood viscosity and blood lipid, such as cholesterol, triglyceride, high density protein, low density protein and hematocrit on CVD have been discussed (Collaboration et al., 2007; Apostolidis and Beris 2016; Vasquez et al., 2019; Cekirdekci and Bugan 2020; Engin and Güvenç 2020). Undoubtedly, these research results have played an important role in the understanding, diagnosis, treatment and prevention of CVD. Of all the risk factors, blood pressure is the greatest concern. Although the incidence of hypertension is high, our understanding of its pathogenesis is still incomplete (Wu 2021). Blood supply disorders can cause changes in blood pressure, and blood supply to the brain, heart and other organs is correlated with their physiological parameters. As CCVD is a systemic, complex and multi-factorial disease that develops over time, risk factors for CCVD need to be considered in terms of more than a single physiological variable as there is a lack of consensus on several topics, including the existence of the J-curve (Vokó et al., 1999; Malyszko et al., 2013; Verdecchia et al., 2017; Yin et al., 2021). Risk factors for CCVD can only be predicted by a small number of physiological parameters such as blood pressure, vascular stiffness, and blood lipids. Due to differences among individuals, it is difficult to use a single physiological parameter to accurately predict the incidence and mortality of CCVD. Therefore, it is necessary to build a model with multiple risk factors. Fluid-structure interaction (FSI) theory is a powerful tool to build a model that can incorporate multiple-factors considering physiological and functional parameters.

FSI theory has been widely used in the study of human blood circulation, especially in the field of hemorheology. There has been great interest in recent research on the effects of vascular elasticity on the distribution of blood flow velocity, wall shear stress and flow velocity (Coccarelli et al., 2021; Chalons et al., 2022), including the effects of different geometric structures (Corti et al., 2020), external conditions (Nardinocchi et al., 2005; Misra et al., 2018), multi-scale factors (Qohar et al., 2021), fluid properties (Shinde et al., 2022) and states (Younis and Berger 2004). There have been many studies on fluid-structure interaction models of blood and arteries, such as the Ventricular-Arterial Coupling model (Pagoulatou et al., 2021), Direct Eulerian Generalized Riemann Problem scheme (Sheng et al., 2021), and Hybrid Windkessel-Womersley coupled model (Aboelkassem and Virag 2019), among others. The above studies are based on elastic cavity theory, which considers the contribution of arteries to dissipation energy, and have primarily generated hemorheological parameters. However, the strain energy of elastic arteries has not been considered as the source of power for blood flow. Because these findings have been difficult to describe in terms of clinically accepted CVD risk factors, they have not been incorporated into clinical practice.

In summary, morbidity and mortality of ischemic or hemorrhagic CCVD are mainly characterized by abnormal blood supply, and blood supply to organs should be a key control quantity for chronic diseases. The availability of adequate nutrients and oxygen to organs depends on the ability of the heart to pump blood and the large vessels to carry it. Unfortunately, the relationship between blood supply to organs and physiological parameters is not well understood since the exact blood flow across a given blood vessel in response to mechanical forces such as systolic/diastolic blood pressure (SBP/DBP) is unclear (Chandran et al., 2012). Therefore, biochemical parameters obtained from routine physical examinations cannot be used to assess blood flow. In order to reduce the incidence and mortality of CCVD, it is necessary to further understand the relationship between physiological parameters and blood supply. Whether disruption of blood supply causes changes in physiological parameters or vice versa is a controversial topic that merits further study.

Our objectives were as follows: firstly, confirm that the active, deformed elastic aorta stores deformation energy, and establish a quantitative relationship between strain energy and functional and structural parameters. Secondly, based on fluid-structure interaction theory, develop a mathematical model of the effect of coupling multiple physiological parameters to blood supply. Disruption of blood supply can cause changes in physiological parameters, and vice versa. This model can be used to evaluate human blood flow using biochemical parameters obtained from routine physical examinations, and can address some controversial problems in the prevention, diagnosis and targeted treatment of CCVD. By contrast with meta-analysis methods, this model describes the relationships between multiple physiological parameters and blood supply to organs. Transforming this theoretical framework into clinical research will provide new evidence for an in-depth knowledge of human blood circulatory physiology.

## 2 Materials, test methods and experimental results

The purpose of this study is to overcome the barrier between biomechanics and clinical practices in the understanding of blood circulation and CVD and build a multi-parameter coupling model with clinical medicine as the background. Therefore, the physiological parameters that can be tested in clinical medicine were selected in the study.

Past studies have shown that compared with brachial blood pressure (BP), central BP is a more accurate predictor of cardio-cerebrovascular risk (Picone et al., 2021), and brachial BP measurements may lead to a 28% misdiagnosis rate in patients (Roman et al., 2007). In this study, the human aorta was used as research object. The aorta of porcine was used as the *in vitro* experimental materials since the aortic mechanical properties and structures of porcine are very similar to that of human beings (Suzuki et al., 2011; Fung 2013; Mathern et al., 2022). Expansion tests and biaxial tensile tests were conducted under different loads. The expansion tests used normal porcine physiological BP and heart rate, 50/120 mmHg and 80 beats/min (Gladczak et al., 2013; Lelovas et al., 2014), respectively. Collection of and experiments on porcine artery tissue were approved by the Ethics Committee of Kunming University of Science and Technology.

### 2.1 Expansion and extension test

**FIGURE 1:**
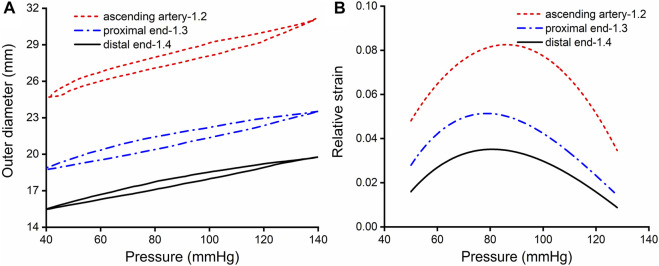
Results of expansion and extension tests. **(A)** Outer diameter-pressure curves of the aorta, with an *in vivo* axial stretch ratio of 1.2, 1.3 and 1.4 for the ascending, proximal descending and distal descending aorta, respectively. **(B)** Relative strain-pressure curves of the ascending, proximal descending and distal descending aorta with an axial *in vivo* stretch ratio of 1.2, 1.3 and 1.4.

The aim of the experiment was to obtain the relative strain of the aorta. Ten porcine aortic arteries were collected from a slaughterhouse in Chenggong, China, as soon as the pigs were sacrificed, and the aortic tissues were transferred to the laboratory. Connective tissues and fat were removed from around each artery. Then, the arteries were divided into ascending, descending proximal, and distal segments, each with a length of 4 cm. Expansion experiments were performed on the tubular artery segments using a dynamic mechanical tester (Electroforce, 5500, TA Instruments, United States). The tubular artery segments were stretched axially to recover their *in vivo* dimensions, with the longitudinal stretch ratio λ_z_ = 1.2, 1.3, 1.4 for the ascending, proximal descending, and distal descending arteries, respectively, according to the results reported by Han and Fung (Han and Fung 1995). Prior to each test, the diameter of the unstressed artery was measured with a digital caliper. The arteries were then dilated with distilled water to simulate physiological and pathological blood pressure. A pressure sensor and laser rangefinder were used to record the relationship between the internal pressure and the external diameter of the artery, which was used to calculate the relative circumferential strain of the aorta during diastole and systole. In clinical practices, the aortic outer diameters can be easily and accurately measured, and then the relative circumferential strain can be calculated. For the convenience of clinical application, engineering strain is used in this paper. The experimental results were shown in Figures 1A,B.

### 2.2 Biaxial planar test

**FIGURE 2:**
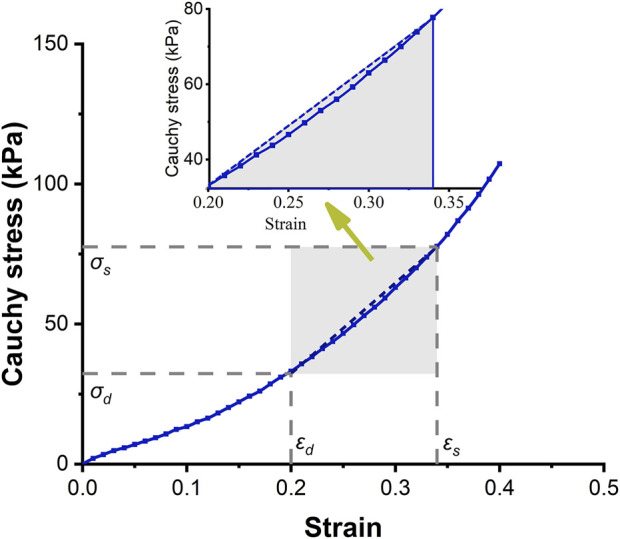
Circumferential stress-strain curve of the ascending aorta. The solid-dotted line represents the nonlinear stress-strain curve, and the dashed line represents a linear approximation.

The purpose of the experiment was to obtain the stress-strain curve of the aorta, which furthermore to deduce the equations of strain energy. After the expansion tests, four square (13 × 13 mm) arterial specimens were cut with a customized cutter to ensure size consistency of sample cut from the artery segments. The edges of the square specimens were parallel with the longitudinal and circumferential orientations. Biaxial tests were performed using a commercial tensile testing machine (Cell Scale, Biomaterials Testing, Waterloo, Canada). The specimens were mounted on the biaxial test system by four bio-rakes and stretched to 1.4× the initial length of the specimen, as the strain in the artery wall was less than 0.4× that under physiological conditions. Planar stretch tests were controlled by displacement in order to reduce creep, and samples were stretched slowly (strain rate of 0.3 per minute). The specimens were preconditioned by ten loading and unloading cycles to obtain stable strain-stretch data, and the last cycle was chosen for analysis. According to the law of Laplace, the range and magnitude of stress and strain were estimated according to the corresponding diameter of the artery segment and physiological blood pressure range. The stress and strain curves of the artery specimens were fitted by an exponential function with a goodness of fit *R* ^2^ ≥ 0.99. According to the paired Student’s *t* test, no statistically significant difference was found between linear and nonlinear approaches (*p* = 0.993, > 0.05). Therefore, the stress-strain behavior of the aortic tissues within the physiological range of BP can be approximated by the linear method. The experimental results were shown in Figure 2.

### 2.3 Experimental platform for simulating the cardiovascular system

**FIGURE 3:**
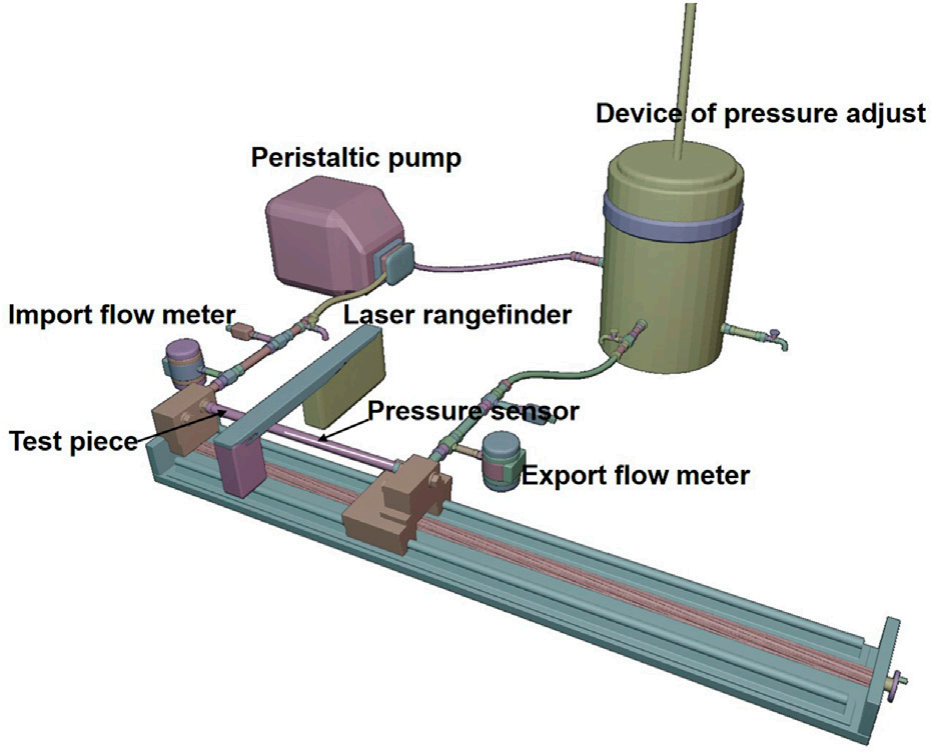
Cardiovascular system experimental simulation platform.

**FIGURE 4:**
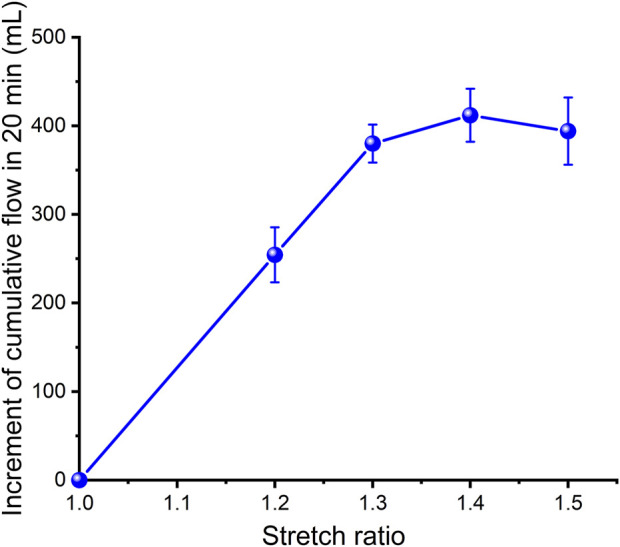
Increment in cumulative flow over 20 min with different stretch ratios for porcine aorta.

In order to estimate the strain energy stored in the aorta and show its relationship with blood flow, a set of closed cardiovascular test simulation systems was designed (Figure 3, China patent number: ZL 201911070403.2). This system consists of a peristaltic pump, extensible elastic tubes, a sealing tank, a pressure adjustment device, a pressure sensor (Millar Mikro-Cath 825-0101, United States), a laser rangefinder (Mitutoyo LMS-503S, United States) and two flow meters. This device can be used to measure changes in the volume of fluid flowing through an elastic tube at different stretch ratios, internal pressures, fluid media and materials, so as to show that the deformed elastic tube has the ability to store strain energy that can be converted into mechanical work to promote fluid flow. The results were reproducible. The experimental flow values were recorded within 20 min and were shown in Figure 4. Although the cross-sectional area of the specimen decreased during stretch, the flow volume increment increased, indicating that the *in vivo* descending artery stored strain energy, which could explain the increase in fluid flow.

### 2.4 Statistical analysis

Statistical analyses were performed by SPSS 26 and Microsoft Excel statistical packages. A paired 2-tailed Student’s *t* test was used to compare the variance of linear and nonlinear approaches to analysis of stress-strain data within the physiological range of BP, with the significance level set to *p* < 0.05. The experimental results are reported as mean ± standard error.

## 3 Strain energy density increment

### 3.1 Strain energy density increment in the aorta

**FIGURE 5:**
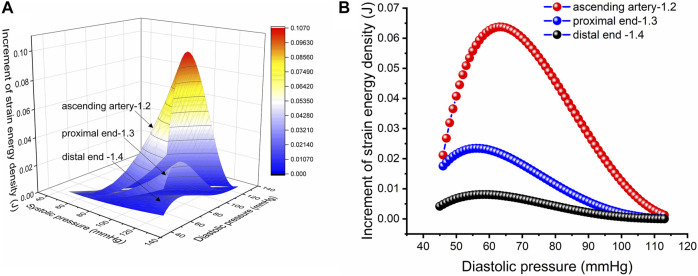
Strain energy density increment per unit length. **(A)** Increment in strain energy density per unit length of the aorta with an *in vivo* axial stretch ratio of 1.2, 1.3 and 1.4 for the ascending, proximal descending and distal descending arteries, respectively. **(B)** Increment in strain energy density per unit length upon *in vivo* axial stretch for the ascending, proximal descending and distal descending arteries, respectively, with an SBP of 113 mmHg.

It is a nature phenomenon that elastic arteries contribute to blood flow. There are few literatures that quantify the contribution of elastic vessels to blood flow, especially the contribution of functional and structural parameters of aorta to blood supply. This research gap was the basis of the innovation of this paper. In order to facilitate clinical applications, the strain energy density increment (SEDI) of the aorta is described by physiological variables that can be measured during clinical practice. Previous studies have shown that despite the viscoelasticity of the elastic aorta, circumferential mechanical properties of the aorta can be approximated by linear elasticity over the physiological range (see Figure 2). As the axial elastic modulus is much larger than the circumferential modulus, and the aorta is constrained by surrounding soft tissues, and there is little longitudinal stretching of the vascular wall, the influence of axial deformation can be ignored (Bergel 1961). The elastic properties of blood vessels are therefore primarily related to circumferential parameters (Bergel 1961). As the ratio of radius to wall thickness of the ascending artery is 10.2 (Mensel et al., 2014; Mensel et al., 2016), the ascending aorta was regarded as a thin-walled cylinder. According to the circumferential stress-strain relationship, the area enclosed by the dashed line in Figure 2 is the linear strain energy density. The SEDI (*∆u*) between the systolic and diastolic periods can be approximated as:

$$\Delta u=\left(\sigma_{s}-\sigma_{d}\right)\left(\varepsilon_{s}-\varepsilon_{d}\right)/2$$ (1)

or

$$\Delta u=\varphi^{2}{\left(\alpha_{s}p_{s}-\alpha_{d}p_{d}\right)}^{2}/8E$$ (2)

in which

$$E=Dp/2h\varepsilon_{\theta },\sigma_{i}=\varphi p_{i}D_{i}/2h_{i},i=s,d$$ (3)

where *p*_*s*_, *p*_*d*_ and *E* (Chandran et al., 2012) represent systolic blood pressure (SBP), diastolic blood pressure (DBP), stiffness of the aorta (circumferential elastic modulus), respectively, *φ* is defined as the expansion constraint coefficient, *D, p, ε*_*θ*_ and *h* are inner diameter, mean blood pressure (MBP) ( $p=\left(2p_{d}+p_{s}\right)/3$ ), relative circumferential strain and wall thickness, respectively. $\;\varepsilon_{\theta }=\varepsilon_{s}-\varepsilon_{d}=\left(D_{s}-D_{o}\right)/D_{o}\;-\left(D_{d}-D_{o}\right)/D_{o}\;=\left(D_{s}-D_{d}\right)/D_{o}\;$ , $\alpha_{s}=D_{s}/h_{s}-2$ , $\alpha_{d}=D_{d}/h_{d}-2$ , where *D*_*s*_ *, D*_*d*_ *, h*_*s*_ and *h*_*d*_ are the outer diameters and wall thicknesses of the aorta in systole and diastole, respectively. *D*_*o*_ is initial outer diameter of aorta. $\sigma_{i}$ is the circumferential stress in systole and diastole. It should be noted that *φ* can also be calculated from artery compliance. It can be seen that the SEDI is positively correlated with the squared value of pulse pressure, and negatively correlated with the circumferential elastic modulus. The above formula can be applied to not only humans, but also other mammals. Figure 5A represents the relationship between SEDI and DBP/SBP in porcine thoracic artery. Figure 5B shows the variation of the SEDI in the distal aorta as a function of diastolic pressure at a systolic pressure of 113 mmHg. It can be seen that the SEDI for the proximal artery is higher than that for the distal artery, indicating that the proximal artery has a higher elastic modulus. Compared with other artery segments, the ascending aorta stores the most strain energy and exhibits the highest elasticity.

### 3.2 Nonlinear and linear approximations of strain energy density in the ascending artery at physiological blood pressure

**FIGURE 6:**
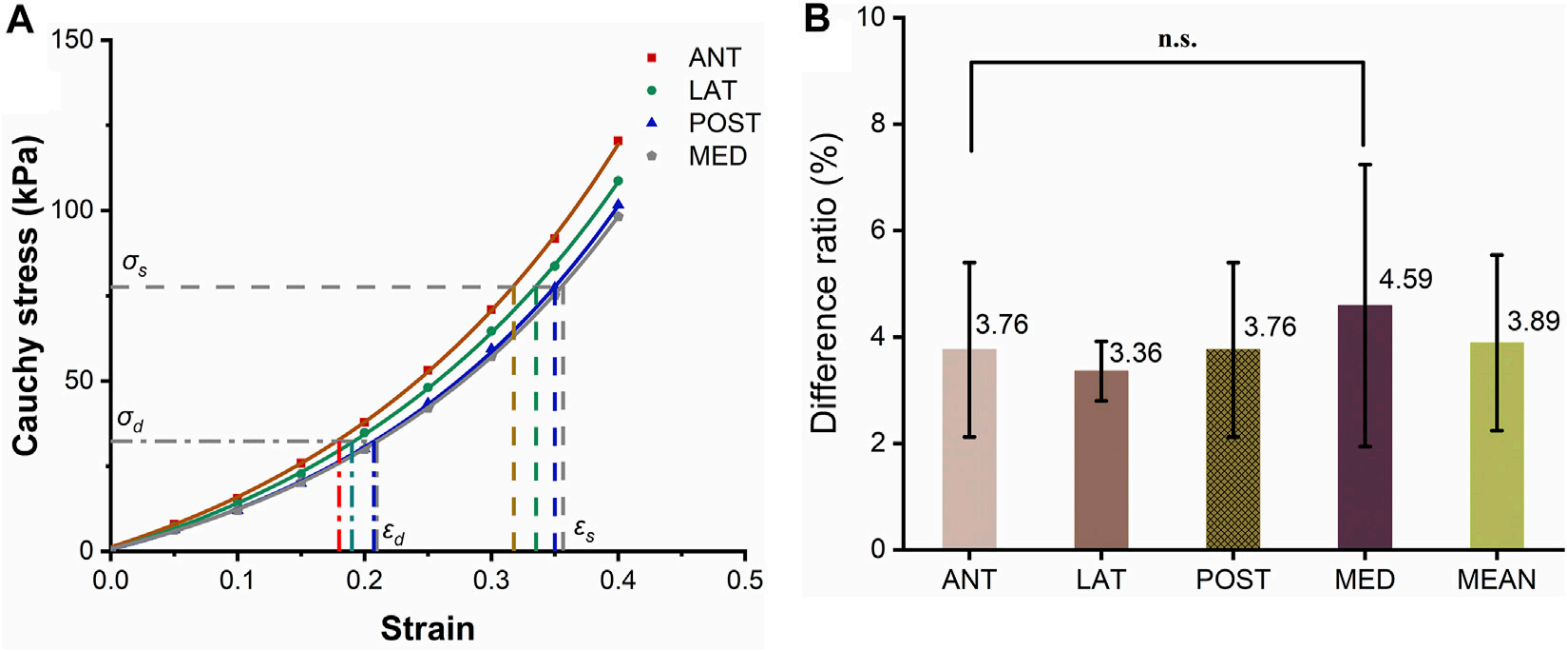
Circumferential stress-strain curves of the ascending artery. **(A)** Stress-strain curves of the anterior (ANT), lateral (LNT), posterior (POST) and medial (MED) quadrants around the circumference of the artery. The horizontal and vertical lines represent the values of stresses and strains calculated using a physiological range of 50/120 mmHg BP for pigs. **(B)** Histogram of the differences in strain energy within the physiological range estimated by linear and integral approximations. The five columns represent the ratio of the linear to integral estimates of stress-strain area for the anterior (ANT), lateral (LNT), posterior (POST) and medial (MED) quadrants and the mean difference, with the values marked above each column. The label n.s. indicates no significant difference among the four quadrants.

Figure 2 shows the circumferential stress-strain curve of porcine ascending artery within the physiological range of BP (50/120 mmHg). The area enclosed by the solid line is the non-linear strain energy density, while that within the dashed line is the linear strain energy density. In the case of the porcine aorta, the difference in strain energy density between the linear and nonlinear values was only 3.8 ± 91.65% (Figures 6A,B). Therefore, linear strain energy density is considered to be an accurate estimate of the nonlinear strain energy density at physiological BP.

## 4 A new fluid-structure coupling model of human blood circulation

### 4.1 Valid base assumptions

In addition to satisfying the basic assumptions of Section 3, blood flow in the aorta was assumed as laminar flow. In the human aorta, the amplitude of Reynolds number *Re* is calculated to be about 1,500, far below the critical value of 2,100. There is no experimental evidence of sustained turbulence in human blood circulation so far. In most of the cardio-vascular cycle, blood flow in the circulatory system can be set as laminar flow. Under special circumstances, some disturbance of blood flow in the aortic root and pulmonary trunk occur as the heart contacts to its peak. Turbulent or highly disturbed flow would occur in the posterior side of the diseased valve, or the stenosis of the blood vessel, or the implanted mechanical devices (Chandran et al., 2012).

### 4.2 The relationship between physiological parameters and blood supply

The relationship between physiological parameters and blood supply was studied. Herein, physiological parameters refer to functional and structural parameters of the aorta, hemorheological properties, blood lipid, pulse, and heart rate.

The kinetic energy unit weight of total blood flow (Shandong Institute of Technology and Northeast Electric Power University 1979) is

$$k=\eta V^{2}/2g$$ (4)

where *η* is the kinetic energy correction factor. For viscous fluid laminar flow *η* = 2. The increment in kinetic energy unit weight is $\Delta K=\Delta V\rho gV^{2}$ , where ΔV is the increment in volume (Zhang and Yang 1996). As cross-sectional area changes ( ΔA ), the increment in kinetic energy unit length is

$$\Delta K=\Delta A\rho gV^{2}=\Delta A\gamma V^{2}$$ (5)

where ρ is blood density and γ is the per-unit-volume weight. In diastole and systole, the increment in flow area ΔA is

$$\Delta A=\varphi \left(D_{s}p_{s}/h_{s}-D_{d}p_{d}/h_{d}\right)A_{f}/E$$ (6)

Thus, the increment in blood kinetic energy unit length ΔK in diastole and systole is

$$\Delta K=\varphi \gamma A_{f}\left(\alpha_{s}p_{s}-\alpha_{d}\sigma_{d}\right)V^{2}/E$$ (7)

The dissipated energy increment during fluid flow $\Delta w_{\tau }$ (the increment in work done by blood and wall shear stress) (Zhang and Yang 1996) is

$$\Delta w_{\tau }=-\gamma \lambda \left(D_{s}-D_{d}\right)K^{2}V^{2}$$ (8)

where *λ* is the friction loss factor of the elastic vessel (which is related to flow state and blood viscosity, lipid content, and velocity) for laminar flow, λ=64/Re , Re=VD/μ , in which *Re* is the Reynolds dimensionless coefficient, *μ* is blood viscosity, and *K* is a coefficient. According to the Hamilton principle for fluid-structure coupling (Shandong Institute of Technology and Northeast Electric Power University 1979; Zhang and Yang 1996), the functional χ of the constructed aortic vessel per pulse unit length can be represented as follows:

$$\chi =\pi D^{2}\varphi \gamma \left(\alpha_{s}p_{s}-\alpha_{d}\sigma_{d}\right)V^{2}/4E-\pi Dh\varphi^{2}{\left(\alpha_{s}p_{s}-\alpha_{d}\sigma_{d}\right)}^{2}/8E-\pi \left(D_{s}-D_{d}\right)\varphi \gamma \lambda DK^{2}V^{2}$$ (9)

where *V* is the flow distance over one pulse through the cross section of the artery per unit weight of blood, which is defined as the characteristic velocity. According to the Hamilton principle of fluid-structure coupling, *V* can be obtained by the variational operation of *D*_*s*_ and *D*_*d*_ *.* We have:

$$V=\sqrt{\frac{\varphi h}{D\gamma \left(1+\beta \right)}\left(\alpha_{s}p_{s}-\alpha_{d}p_{d}\right)}$$ (10)

in which

$$\beta =\frac{4E\lambda \left(h_{d}-h_{s}\right)}{D\left(p_{s}-p_{d}\right)\varphi }$$ (11)

where β is defined as the coupling energy dissipation coefficient and is related to fluid viscosity, blood lipid, vascular geometry, pulse pressure, and vascular stiffness. Therefore, the average blood flow *Q* of each pulse through the cross section is:

$$Q=Q_{0}+\Delta Q=\left(1+\varphi \frac{Dp}{Eh}\right)A_{f}\sqrt{\frac{\varphi h}{D\gamma \left(1+\beta \right)}\left(\alpha_{s}p_{s}-\alpha_{d}p_{d}\right)}$$ (12)

where *A*_*f*_ is the blood flow area of the aorta. The average flow volume $\bar{Q}$ per minute through the cross section of the vessel is:

$$\bar{Q}=n\left(1+\varphi \frac{Dp}{Eh}\right)A_{f}\sqrt{\frac{\varphi h}{D\gamma \left(1+\beta \right)}\left(\alpha_{s}p_{s}-\alpha_{d}p_{d}\right)}$$ (13)

where n is the pulse frequency. This model describes the effect of multiple physiological variables on blood supply. To illustrate applications of the above formulas, four examples are provided.

1) If central BP is set to 80/110 mmHg at rest and 80/120 mmHg in the normal state, the area of the ascending artery expands by 3% and 4%, respectively (Lu et al., 2017). Taking *E* = 3.5 × 105 Pa, *Re*= 1,000, *D* = 32.0 mm, *h* =1.46 mm (Mensel et al., 2014; Mensel et al., 2016; Lu et al., 2017), *φ* = 0.0439/0.0565 (rest and normal states), *β* = 0.375/0.290 (rest and normal states), and *K* = 1 (laminar flow), average blood flow volume calculated by Eq. 13 is 5.23 and 7.95 L/min at rest and in the normal state, respectively. These values are consistent with clinical measurements.
2) According to clinical statistical studies (Schutte et al., 2005), the physiological parameters of healthy humans are: BP of 123/72 mmHg, heart rate of 64.8 bmp, and arterial compliance of 1.78 ml/mmHg, while for hypertensive patients, the corresponding values are 156/91 mmHg, 69.1 bmp, and 1.65 ml/mmHg. Ceteris paribus, the DBP of hypertensive patients calculated by Eq. 13 is 94.3 mmHg, resulting in a deviation of 3.6% from clinical data.
3) It has been reported that there is a significant difference in the relative risk of hemorrhagic stroke and different types of ischemic stroke with BP higher than 110/180 mmHg (28.83% vs 9.56%) (Wu et al., 2012). According to Eq. 13, within this range, blood flow increased by over 31% compared with that of healthy people. This data does not reflect the risk of hemorrhagic stroke, but does indicate the likelihood of hemorrhagic stroke. In this situation, more blood is retained in the cerebrovascular system, which leads to increased cerebral BP and risk of brain vessel rupture.
4) Changes in SBP and pulse pressure (PP) in diastolic hypertension patients suggest a 12% increase in the incidence of CVD events at the end of 2 years in patients with lower DBP (75 mmHg) compared with patients with higher DBP (95 mmHg) when SBP was 160 mmHg for both groups (Amery et al., 1985; Staessen et al., 1997; Wang et al., 2000). According to Eq. 13, blood flow volume in the low DBP group (75 mmHg) increased by 23% compared with the high DBP group (95 mmHg), leading to an increase in hemorrhagic stroke.

## 5 Discussion

The model established in this paper is a new understanding of blood circulation. It is emphasized that the heart pumps blood at an initial rate to provide initial kinetic energy for circulation, then, the potential energy (i.e. strain energy) of arterial vessels drives blood flow, and the work done by the shear force on the blood vessel wall is the dissipated energy, which forms the whole blood circulation with three parts. The current FSI models about blood flow and arteries are based on Windkessel model or modified model (Aboelkassem and Virag 2019; Pagoulatou et al., 2021; Chalons et al., 2022). Different from the Windkessel model, the new model shows that in addition to the power provided by the heart for blood circulation, strain energy stored in deformed arterial vessels *in vivo* can propel blood flow. In the study of hemorheology, the influence of blood flow field, state, velocity and shear stress caused by vasculopathy on vascular wall has been focused on, especially the change of flow field and stress field when plaque, atherosclerosis, lipid accumulation and calcification occur in vascular wall (Boujena et al., 2014; Lopes et al., 2020; Lipp et al., 2020). While the new model focuses on the correlation with average flow and physiological parameters, which is more significant for clinical medical applications. The model of multi-parameter blood flow presented in this paper is an approximate analytical solution of average velocity rather than numerical solution. The relationship between this model and CCVD is discussed as follows.

### 5.1 Mechanical mechanism of the J-curve

**FIGURE 7:**
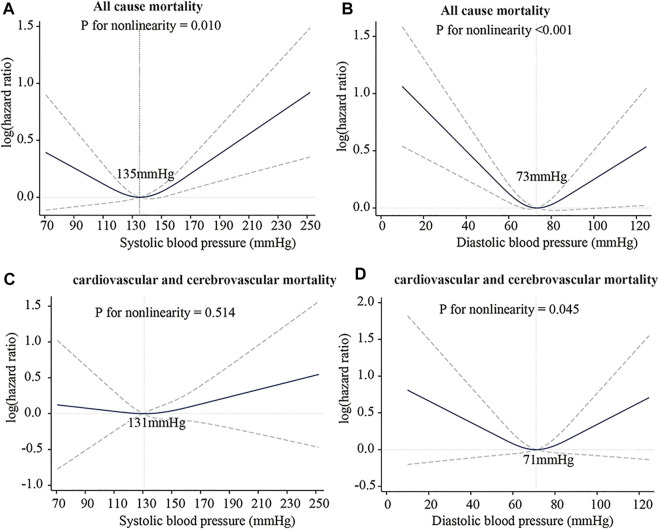
J-Curve of the association between blood pressure and risk of death. **(A)** SBP: all-cause mortality. **(B)** DBP: all-cause mortality. **(C)** SBP: cardiovascular and cerebrovascular mortality. **(D)** DBP: cardiovascular and cerebrovascular mortality. International Journal of General Medicine 2021:14 5039-5049 Originally published by and used with permission from Dove Medical Press Ltd.

The J-curve has existed in clinical practice for decades and has been well known to clinical medicine physicians and scholars. The J-curve represents the relationship between blood pressure and mortality of CCVD, the risk rate and mortality of CCVD increased when DBP/SBP is too high or low, as shown in Figure 7 (Yin et al., 2021). Whether the J-curve exists is the focus of debate among medical experts (Vokó et al., 1999; Malyszko et al., 2013; Verdecchia et al., 2017; Yin et al., 2021). Our results support the existence of the J-curve (Figure 5B).

Strain energy provided by arteries can promote blood flow. In comparing the SEDI curve with the J-curve, it was found that SEDI is negatively associated with the risk of cardiovascular disease (CVD). At a given SBP (from Figure 5B), when DBP is too high or too low, the circumferential relative strain is small (see Figure 1B), so does the SEDI stored in the aorta (see Figure 5B). This indicates that inadequate strain energy in the aorta results in insufficient energy to promote blood flow, thus impairing blood supply to target organs and increasing the risk of CVD. However, for the same SBP, the risk of CVD caused by excessively low DBP is lower than that caused by high DBP. This is consistent with the J-curve in that the risk and mortality of CVD increases when DBP is too high or low. This means that lower BP is not always better for treatment of hypertension. Only when the SBP and DBP are within a defined range can the aorta provide the optimal strain energy and promote adequate flow.

**FIGURE 8:**
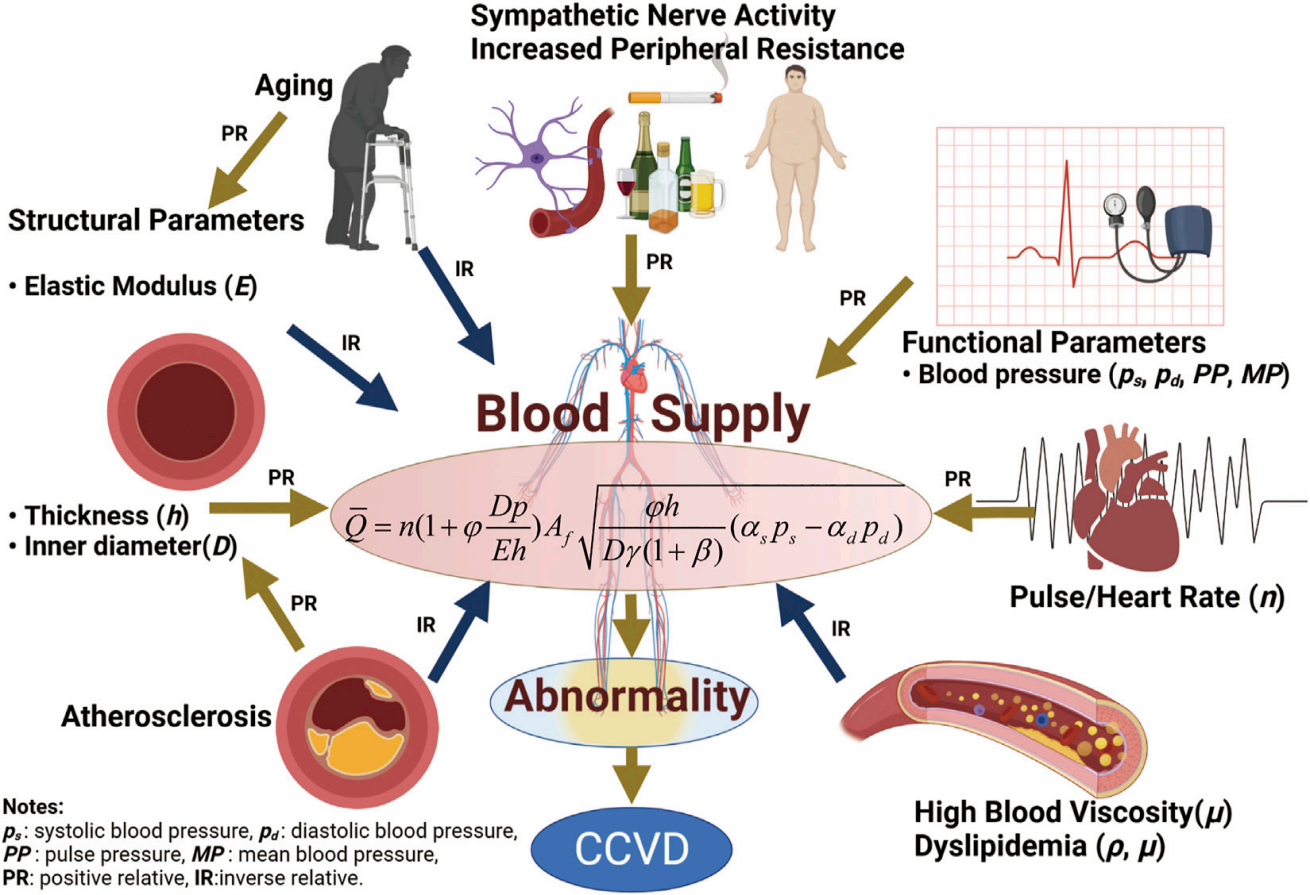
Relationship between blood flow and physiological variables.

The relationship between blood flow and several physiological variables is described in Eq. 13 and shown in Figure 8.

### 5.2 Structural and functional parameters of the aorta and their effects on blood supply

Aortic mean blood flow is closely related to structural and functional parameters that can be measured easily in clinical practice, so their relationship to CCVD has been a topic of intense research. While previous studies have qualitatively described a bidirectional correlation between structural and functional parameters of the vasculature, the equation proposed in this paper quantitatively describes the relationship between them and how it is altered by changes in blood supply.

### 5.1.1 Vascular stiffness and blood supply

As can be seen from Eq. 12, without considering BP, *Q*_*0*_ is only related to the geometric size of the aorta, while *ΔQ* is not only related to geometric size, but also negatively related to *E*. With an increase in *E* resulting from, e.g., calcification, high cholesterol level, lipid deposition, plaque or atherosclerosis in the artery intima, vascular smooth muscle cell senescence, or increased oxidative stress in the arteries, the contribution of *ΔQ* to total blood supply decreases. If the value of arterial stiffness is sufficiently high, the contribution of *ΔQ* to total blood supply is minimal. In this case, blood supply is only related to PP and aortic geometry. As the media-intima thickness (MIT) of the aorta increases, PP inevitably increases, but has little effect on the value of MBP. Therefore, it is clear that increased PP can serve as an indicator of increased aortic stiffness. The results obtained by this model are consistent with current clinical reports (Laurent et al., 2001a, b; Boutouyrie et al., 2002; Laurent et al., 2003; Wu et al., 2012; Ecobici and Stoicescu 2017; Lyle and Raaz. 2017; Virdis 2018; Tomiyama 2020).

#### 5.1.2 Vascular geometry and blood supply

It can be seen from Eq. 13 that vascular geometry is positively correlated with blood supply. The geometry of the arteries changes with age. Both the inner and outer diameters increase and the MIT thickens with age, but the increase in outer diameter is greater than that in inner diameter, indicating an increase in wall thickness (Jadidi et al., 2019). In addition, the incidence of intima calcification, high cholesterol, lipid deposition, plaque or atherosclerosis, senescence of vascular smooth muscle cells, and increased arterial oxidative stress also contribute to MIT thickening and an inevitable decrease in blood supply, although the nervous system compensates by raising BP to ensure adequate blood flow (McEniery et al., 2007; Jadidi et al., 2020).

#### 5.1.3 Functional parameters of the aorta and blood supply

It can also be seen in Eq. 13 that $\bar{Q}$ is a monotonically increasing function for SBP and MBP, that is, when SBP and MBP are excessively high, blood supply increases greatly. Excessive blood supply can cause damage to organs, resulting in fatal diseases such as hemorrhagic stroke, cognitive impairment, heart failure, CKD, and aneurysm, among others (see example 3 in Section 4). Clinical illnesses such as metabolic disorders, blood sugar disorders and obesity increase the demand for blood, resulting in increased SBP and MBP, which eventually leads to hypertension. Long-term high PP or high MBP can lead to excessive blood volume in the vascular system and increase pressure in the arteries, resulting in blood vessel rupture and bleeding (i.e., hemorrhagic stroke) in weak vessels (see example 4 in Section 4). Increased SBP and MBP are related to increased arterial stiffness, changes in geometric parameters, dyslipidemia and high blood viscosity (see Eq. 13). However, a need for increased blood supply can result from a variety of causes, including metabolic disorders, blood disorders, obesity, Marfan’s syndrome, sleep disorders, liver disease, elastic pseudo xanthoma, aneurysm, and emotional stress (e.g., fear, excitement or sadness). SBP and MBP must rise to keep up with the requirements of organs for blood.

DBP is also closely related to arterial stiffness and blood supply, and excessive hypotension would reduce blood supply to organs, leading to increased incidence of ischemic CCVD. In clinical treatment of hypertension, personalized treatment should consider matching arterial structural parameters and functional parameters, blood lipid, and rheological parameters of patients. Blindly lowering BP without considering other parameters will reduce blood supply (see Eq. 13), which can lead to chronic ischemic diseases. In China, the incidence of ischemic diseases (such as coronary heart disease and cerebral infarction) after treatment of hypertensive patients is much higher than that of patients with cerebral hemorrhage (Wu et al., 2012). The question of whether this is caused by lowering BP alone without considering other physiological parameters merits further consideration. In addition, a sudden BP drop deprives the brain and vital organs of oxygen and nutrients and can result in fatal outcomes such as coronary artery disease and cerebral infarction. Therefore, the optimal goal of hypertension treatment (control of functional parameters) should be aimed at ensuring appropriate blood supply to organs. The above factors are described by Eq. 13, in good agreement with clinical studies (Safar 2008; Verdecchia et al., 2012; Wu et al., 2012; Malyszko et al., 2013).

### 5.3 Dyslipidemia, high blood viscosity and blood supply

It can be seen from Eq. 11 that the coefficient *β* can be affected by many factors. Only the effects of dyslipidemia and blood viscosity are discussed here. Increased blood viscosity and dyslipidemia result in a higher *β* value and lower average flow velocity and blood supply (see Eqs 10, 13. In order to provide sufficient blood supply, BP has to be increased, which leads to chronic hypertension. In addition, acute ischemic diseases are often caused by dyslipidemia, high blood viscosity, and rapid declines in blood flow velocity, and are manifested as a marked decrease in blood supply. These results are in good agreement with clinical studies (Collaboration et al., 2007; Apostolidis and Beris 2016; Vasquez et al., 2019; Cekirdekci and Bugan 2020; Engin and Güvenç 2020).

### 5.4 Heart rate, pulse rate and blood supply

In normal healthy people, the heart rate is equal to the pulse rate. For patients with long-term hypertension, the strain energy stored in elastic arteries decreases as arterial stiffness increases, reducing the blood supply to organs, often accompanied by weight gain and diseases like diabetes and lipid disorders. Insufficient blood supply to organs often occurs in patients with chronic hypertension undergoing treatment. Due to sympathetic regulation of the heart, heart rate can be greater than pulse rate, and blood pumped by the heart cannot in that case be fully transported to organs through the aorta, eventually resulting in atrial fibrillation, where excess blood accumulates in the left atrium, resulting in thrombosis. When the clot breaks off, carotid artery blockage can lead to ischemic stroke. Atrial fibrillation is not only related to heart function and the sympathetic nervous system, but also to the ability of the aorta to supply blood. Proper physical exercise helps to raise the pulse rate and increase blood flow, which is beneficial to patients in a long-term ischemic state. The above discussion is consistent with recent research (Isabelle et al., 2016; Warnert et al., 2016; Campbell et al., 2019; Presa et al., 2020; Tofas et al., 2021; Waclawovsky et al., 2021).

### 5.5 Sympathetic nervous system, peripheral resistance and blood supply

Increased sympathetic activity and poor habits such as lack of sleep, smoking and obesity can lead to increased peripheral resistance and a need for increased blood supply (Seravalle and Grassi 2017; Omboni 2020; Boutouyrie et al., 2021). However, vascular structural parameters cannot rapidly change in response. According to Eq. 13, for average blood flow ( $\bar{Q}$ ), the change of MBP is more obvious than that of PP. Therefore, MBP has to increase to provide sufficient blood supply, that is, DBP has to increase while PP decreases, resulting in a clinical diagnosis of isolated diastolic hypertension. For instance, if BP is 120/95 mmHg (SBP/DBP), it can be seen from Eq. 13 that blood flow volume would be reduced by 18.2%. Long-term sustained isolated diastolic hypertension causes insufficient blood supply to the body, which is clinically manifested as heat discomfort, dizziness, listlessness of spirit, and weakness of limbs, among other symptoms (Chrysant 2020; Monzo et al., 2021).

### 5.6 Aging and blood supply

Aging-related changes in aortic structural parameters (such as increased stiffness, thickened MIT, increased outer diameter and decreased blood flow area) result in decreased blood flow (Chang et al., 2017; Ferrara et al., 2018; Jadidi et al., 2021). In early stages, MBP and PP rise in order to ensure adequate blood supply, with clinical manifestations mostly diagnosed as isolated systolic hypertension (Ferrara et al., 2018; Yu and Zhang 2021). With aging, aortic stiffness increases, which along with less physical activity and lower demand for blood supply leads to decreased MBP (Mikael et al., 2017; Boutouyrie et al., 2021; Conell et al., 2021) that mainly manifests as decreased DBP and increased PP.

### 5.7 Expansion constraint coefficient

Elastic vessels cannot dilate freely due to constraints imposed by surrounding soft tissues. At the same time, the influence of variables such as aging and other factors cause soft tissue constraints on blood vessels to change. Here, $\varepsilon_{Ap}$ *=* φpD/Eh *,* in which $\varepsilon_{Ap}$ is the circumferential aortic cross section area strain. It can be seen that when $\varepsilon_{Ap}$ is constant, φ is positively correlated with stiffness and wall thickness of blood vessels, and negatively correlated with MBP and inner diameter of arteries, and φ can also be calculated from artery compliance.

## 6 Conclusion

If the heart is one “pump” for blood flow, the aorta is another “pump” that deforms to store strain energy that is converted into mechanical work to force blood through arteries. Blood supply abnormalities are the root cause of morbidity, death and disability associated with CCVD, and those caused by arteriosclerosis precede the appearance of organ diseases. The optimal goal of hypertension treatment, control of functional parameters, should be to ensure adequate and appropriate blood supply to organs. This study quantitatively presents the relationships between blood flow volume and various physiological variables and between strain energy and macro-vascular parameters. Disruption of blood supply can be caused by changes in physiological parameters, and vice versa. The present model can also explain pathogenesis and development of chronic conditions such as CCVD from the perspective of clinical medicine. Changes in one physiological variable will affect many others. To reduce the incidence of CCVD, more attention must be paid to management of the health of the aorta. This study can help to guide accurate prediction, prevention, diagnosis and clinical treatment of CCVD.

### 7 Limitations

Although the results calculated from the quantitative mathematical model are in good agreement with the epidemiology study results, further clinical verification is still needed. In the derivation of the expression of SEDI of the aorta, we followed the assumptions of linear elasticity in the physiological range, the influence of axial deformation was ignored.

## Data Availability

The original contributions presented in the study are included in the article/Supplementary Material, further inquiries can be directed to the corresponding author/s.

## References

[B1] Aboelkassem Y. Virag Z. (2019). A Hybrid windkessel-womersley model for blood flow in arteries. J. Theor. Biol. 462, 499–513. 10.1016/j.jtbi.2018.12.005 30528559

[B2] Amabili M. Balasubramanian P. Bozzo I. Breslavsky I. D. Ferrari G. (2019). Layer-specific hyperelastic and viscoelastic characterization of human descending thoracic aortas. J. Mech. Behav. Biomed. Mat. 99, 27–46. 10.1016/j.jmbbm.2019.07.008 31330442

[B3] Amery A. Birkenhäger W. Brixko P. Bulpitt C. Clement D. Deruyttere M. (1985). Mortality and morbidity results from the European working party on high blood pressure in the elderly trial. Lancet 15, 1349–1354. 10.1016/s0140-6736(85)91783-0 2861311

[B4] Apostolidis A. J. Beris A. N. (2016). The effect of cholesterol and triglycerides on the steady state shear rheology of blood. Rheol. Acta 55, 497–509. 10.1007/s00397-015-0889-0

[B5] Bergel D. H. (1961). The dynamic elastic properties of the arterial wall. J. Physiol. 156, 458–469. 10.1113/jphysiol.1961.sp006687 16992076 PMC1359897

[B6] Boujena S. Kafi O. Khatib N. E. (2014). A 2D mathematical model of blood flow and its interactions in an atherosclerotic artery. Math. Model. Nat. Phenom. 9, 46–68. 10.1051/mmnp/20149605

[B7] Boutouyrie P. Chowienczyk P. Humphrey J. D. Mitchell G. F. (2021). Arterial stiffness and cardiovascular risk in hypertension. Circ. Res. 128, 864–886. 10.1161/circresaha.121.318061 33793325

[B8] Boutouyrie P. Tropeano A. Asmar R. Gautier I. Benetos A. Lacolley P. (2002). Aortic stiffness is an independent predictor of primary coronary events in hypertensive patients: A longitudinal study. Hypertension 39, 10–15. 10.1161/hy0102.099031 11799071

[B9] Brandfonbrener M. Landowne M. Shock N. W. (1955). Changes in cardiac output with age. Circulation 12, 557–566. 10.1161/01.cir.12.4.557 13261308

[B10] Campbell B. C. De, Silva D. A. Macleod M. R. Coutts S. B. Schwamm L. H. Davis S. M. (2019). Ischaemic stroke. Nat. Rev. Dis. Prim. 5, 70–22. 10.1038/s41572-019-0118-8 31601801

[B11] Cekirdekci E. I. Bugan B. (2020). Whole blood viscosity in microvascular angina and coronary artery disease: Significance and utility. Rev. Port. Cardiol. Engl. Ed. 39, 17–23. 10.1016/j.repc.2019.04.008 32156449

[B12] Chalons C. DelGrosso A. Toro E. F. (2022). Numerical approximation and uncertainty quantification for arterial blood flow models with viscoelasticity. J. Comput. Phys. 457, 111071. 10.1016/j.jcp.2022.111071

[B13] Chandran K. B. Rittgers S. E. Yoganathan A. P. (2012). Biofluid mechanics: The human circulation (second edition). Cardiovasc. Eng. Technol. 3, 351–352. 10.1007/s13239-012-0106-6

[B14] Chang C. Y. Chang R. W. Hsu S. H. Wu M. S. Cheng Y. J. Kao H. L. (2017). Defects in vascular mechanics due to aging in rats: Studies on arterial wave properties from a single aortic pressure pulse. Front. Physiol. 8, 503. 10.3389/fphys.2017.00503 28751867 PMC5508003

[B15] Chrysant S. G. (2020). The clinical significance of isolated diastolic hypertension. Postgrad. Med. 132, 624–628. 10.1080/00325481.2020.1788294 32594846

[B16] Coccarelli A. Carson J. M. Aggarwal A. Pant S. (2021). A framework for incorporating 3D hyperelastic vascular wall models in 1D blood flow simulations. Biomech. Model. Mechanobiol. 20, 1231–1249. 10.1007/s10237-021-01437-5 33683514 PMC8298378

[B17] Collaboration P. S. Lewington S. Whitlock G. Clarke R. Sherliker P. Emberson J. (2007). Blood cholesterol and vascular mortality by age, sex, and blood pressure: A meta-analysis of individual data from 61 prospective studies with 55 000 vascular deaths. Lancet 370, 1829–1839. 10.1016/S0140-6736(07)61778-4 18061058

[B18] Conell C. Flint A. C. Ren X. Banki N. M. Chan S. L. Rao V. A. (2021). Underdiagnosis of isolated systolic and isolated diastolic hypertension. Am. J. Cardiol. 141, 56–61. 10.1016/j.amjcard.2020.11.020 33285092

[B19] Corti A. Chiastra C. Colombo M. Garbey M. Migliavacca F. Casarin S. (2020). A fully coupled computational fluid dynamics-agent-based model of atherosclerotic plaque development: Multiscale modeling framework and parameter sensitivity analysis. Comput. Biol. Med. 118, 103623. 10.1016/j.compbiomed.2020.103623 31999550

[B20] Desyatova A. MacTaggart J. Kamenskiy A. (2017). Constitutive modeling of human femoropopliteal artery biaxial stiffening due to aging and diabetes. Acta Biomater. 64, 50–58. 10.1016/j.actbio.2017.09.042 28974476 PMC5682228

[B21] Ecobici M. Stoicescu C. (2017). Arterial stiffness and hypertension–which comes first? Maedica 12, 184–190. 29218066 PMC5706758

[B22] Engin M. Güvenç O. (2020). Investigation of the predictive values of triglyceride/HDL cholesterol ratio and whole blood viscosity with regard to severe peripheral or carotid artery disease in patients scheduled for coronary bypass. Heart Surg. Forum 23, e310–e314. 10.1532/hsf.2991 32524970

[B23] Ferrara A. Totaro P. Morganti S. Auricchio F. (2018). Effects of clinico-pathological risk factors on *in-vitro* mechanical properties of human dilated ascending aorta. J. Mech. Behav. Biomed. Mat. 77, 1–11. 10.1016/j.jmbbm.2017.08.032 28886508

[B24] Fung Y. C. (2013). Biomechanics: Mechanical properties of living tissues. Berlin: Springer Science & Business Media.

[B25] Fung Y. C. Fronek K. Patitucci P. (1979). Pseudoelasticity of arteries and the choice of its mathematical expression. Am. J. Physiology-Heart Circulatory Physiology 237, H620–H631. 10.1152/ajpheart.1979.237.5.h620 495769

[B26] Gasser T. C. Ogden R. W. Holzapfel G. A. (2006). Hyperelastic modelling of arterial layers with distributed collagen fibre orientations. J. R. Soc. Interface 3, 15–35. 10.1098/rsif.2005.0073 16849214 PMC1618483

[B27] Gladczak A. K. Shires P. K. Stevens K. A. Clymer J. W. (2013). Comparison of indirect and Direct blood pressure monitoring in normotensive swine. Res. Vet. Sci. 95, 699–702. 10.1016/j.rvsc.2013.05.013 23790711

[B28] Han H. C. Fung Y. C. (1995). Longitudinal strain of canine and porcine aortas. J. Biomech. 28, 637–641. 10.1016/0021-9290(94)00091-h 7775500

[B29] Hoffman A. H. Teng Z. Zheng J. Wu Z. Woodard P. K. Billiar K. L. (2017). Stiffness properties of adventitia, media, and full thickness human atherosclerotic carotid arteries in the axial and circumferential directions. J. Biomech. Eng. 139, 124501. 10.1115/1.4037794 PMC5686769 28857112

[B30] Holzapfel G. A. Linka K. Sherifova S. Cyron C. J. (2021). Predictive constitutive modelling of arteries by deep learning. J. R. Soc. Interface 18, 20210411. 10.1098/rsif.2021.0411 34493095 PMC8424347

[B31] Idema R. N. Meiracker A. Balk A. Bos E. Schalekamp M. Man A. J. (1994). Abnormal diurnal variation of blood pressure, cardiac output, and vascular resistance in cardiac transplant recipients. Circulation 90, 2797–2803. 10.1161/01.cir.90.6.2797 7994823

[B32] Isabelle M. Chimenti S. Beaussier H. Gransagne D. Villeneuve N. Safar M. E. (2016). SBP, DBP, and pulse blood pressure variability are temporally associated with the increase in pulse wave velocity in a model of aortic stiffness. J. Hypertens. Los. Angel. 34, 666–675. 10.1097/HJH.0000000000000838 26938811

[B33] Jadidi M. Desyatova A. MacTaggart J. Kamenskiy A. (2019). Mechanical stresses associated with flattening of human femoropopliteal artery specimens during planar biaxial testing and their effects on the calculated physiologic stress–stretch state. Biomech. Model. Mechanobiol. 18, 1591–1605. 10.1007/s10237-019-01162-0 31069592 PMC7191998

[B34] Jadidi M. Habibnezhad M. Anttila E. Maleckis K. Desyatova A. MacTaggart J. (2020). Mechanical and structural changes in human thoracic aortas with age. Acta Biomater. 103, 172–188. 10.1016/j.actbio.2019.12.024 31877371 PMC6982607

[B35] Jadidi M. Razian S. A. Habibnezhad M. Anttila E. Kamenskiy A. (2021). Mechanical, structural, and physiologic differences in human elastic and muscular arteries of different ages: Comparison of the descending thoracic aorta to the superficial femoral artery. Acta Biomater. 119, 268–283. 10.1016/j.actbio.2020.10.035 33127484 PMC7738395

[B36] Laurent S. Boutouyrie P. Asmar R. Gautier I. Laloux B. Guize L. (2001a). Aortic stiffness is an independent predictor of all-cause and cardiovascular mortality in hypertensive patients. Hypertension 37, 1236–1241. 10.1161/01.hyp.37.5.1236 11358934

[B37] Laurent S. Katsahian S. Fassot C. Tropeano A. I. Gautier I. Laloux B. (2003). Aortic stiffness is an independent predictor of fatal stroke in essential hypertension. Stroke 34, 1203–1206. 10.1161/01.STR.0000065428.03209.64 12677025

[B38] Laurent S. Tropeano A. Lelouet A. L. Jondeau G. Laloux B. Boutouyrie P. (2001b). Local pulse pressure is a major determinant of large artery remodelling. Clin. Exp. Pharmacol. Physiol. 28, 1011–1014. 10.1046/j.1440-1681.2001.03569.x 11903305

[B39] Lelovas P. P. Kostomitsopoulos N. G. Xanthos T. T. (2014). A comparative anatomic and physiologic overview of the porcine heart. J. Am. Assoc. Lab. Anim. Sci. 53, 432–438. 25255064 PMC4181683

[B40] Lipp S. N. Niedert E. E. Cebull H. L. Diorio T. C. Ma J. L. Rothenberger S. M. (2020). Computational hemodynamic modeling of arterial aneurysms: A mini-review. Front. Physiol. 11, 454. 10.3389/fphys.2020.00454 32477163 PMC7235429

[B41] Lisický O. Hrubanová A. Burša J. (2021a). Interpretation of experimental data is substantial for constitutive characterization of arterial tissue. J. Biomech. Eng. 143, 104501. 10.1115/1.4051120 33973008

[B42] Lisický O. Hrubanová A. Staffa R. Vlachovský R. Burša J. (2021b). Constitutive models and failure properties of fibrous tissues of carotid artery atheroma based on their uniaxial testing. J. Biomech. 129, 110861. 10.1016/j.jbiomech.2021.110861 34775341

[B43] Lopes D. Puga H. Teixeira J. Lima R. (2020). Blood flow simulations in patient-specific geometries of the carotid artery: A systematic review. J. Biomech. 111, 110019. 10.1016/j.jbiomech.2020.110019 32905972

[B44] Lu W. Jia X. Guo W. Liu J. Ge Y. Zhang W. (2017). Aortic lumen diameter change during systolic and diastolic periods: Evaluation with ECG-gated computed tomography. In Chinese. Chin. J. Gen. Surg. 32, 497–500.

[B45] Lyle A. N. Raaz U. (2017). Killing me unsoftly: Causes and mechanisms of arterial stiffness. Arterioscler. Thromb. Vasc. Biol. 37, e1–e11. 10.1161/ATVBAHA.116.308563 28122777 PMC5308873

[B46] Malyszko J. Muntner P. Rysz J. Banach M. (2013). Blood pressure levels and stroke: J-curve phenomenon? Curr. Hypertens. Rep. 15, 575–581. 10.1007/s11906-013-0402-z 24158455 PMC3838583

[B47] Mathern N. Yousefian E. Ridwan H. Nikoubashman O. Wiesmann M. (2022). Comparison of porcine and human vascular diameters for the optimization of interventional stroke training and research. Plos One 17, e0268005. 10.1371/journal.pone.0268005 35503785 PMC9064086

[B48] McEniery C. M. Wilkinson I. B. Avolio A. P. (2007). Age, hypertension and arterial function. Clin. Exp. Pharmacol. Physiol. 34, 665–671. 10.1111/j.1440-1681.2007.04657.x 17581227

[B49] Mensel B. Heßelbarth L. Wenzel M. Kühn J. P. Dörr M. Völzke H. (2016). Thoracic and abdominal aortic diameters in a general population: MRI-based reference values and association with age and cardiovascular risk factors. Eur. Radiol. 26, 969–978. 10.1007/s00330-015-3926-6 26208859

[B50] Mensel B. Quadrat A. Schneider T. Kühn J. P. Dörr M. Völzke H. (2014). MRI-based determination of reference values of thoracic aortic wall thickness in a general population. Eur. Radiol. 24, 2038–2044. 10.1007/s00330-014-3188-8 24816934

[B51] Mikael L. D. R. Paiva A. M. G. D. Gomes M. M. Sousa A. L. L. Jardim P. C. B. V. Vitorino P. V. D. O. (2017). Vascular aging and arterial stiffness. Arq. Bras. Cardiol. 109, 253–258. 10.5935/abc.20170091 28678931 PMC5586233

[B52] Misra J. C. Adhikary S. D. Mallick B. Sinha A. (2018). Mathematical modeling of blood flow in arteries subject to a vibrating environment. J. Mech. Med. Biol. 18, 1850001. 10.1142/s021951941850001x

[B53] Monzo L. Ferreira J. P. Lamiral Z. Bozec E. Boivin J. M. Huttin O. (2021). Isolated diastolic hypertension and target organ damage: Findings from the STANISLAS cohort. Clin. Cardiol. 44, 1516–1525. 10.1002/clc.23713 34523741 PMC8571544

[B54] Nardinocchi P. Pontrelli G. Teresi L. (2005). A one-dimensional model for blood flow in prestressed vessels. Eur. J. Mech. - A/Solids 24, 23–33. 10.1016/j.euromechsol.2004.10.002

[B55] Niestrawska J. A. Viertler C. Regitnig P. Cohnert T. U. Sommer G. Holzapfel G. A. (2016). Microstructure and mechanics of healthy and aneurysmatic abdominal aortas: Experimental analysis and modelling. J. R. Soc. Interface 13, 20160620. 10.1098/rsif.2016.0620 27903785 PMC5134013

[B56] Noble C. Carlson K. D. Neumann E. Dragomir-Daescu D. Erdemir A. Lerman A. (2020). Patient specific characterization of artery and plaque material properties in peripheral artery disease. J. Mech. Behav. Biomed. Mat. 101, 103453. 10.1016/j.jmbbm.2019.103453 PMC6889048 31585351

[B57] Omboni S. (2020). Smoking and hypertension: What is behind the mask? J. Hypertens. Los. Angel. 38, 1029–1030. 10.1097/HJH.0000000000002423 32371790

[B58] Pagoulatou S. Adamopoulos D. Rovas G. Bikia V. Stergiopulos N. (2021). The effect of left ventricular contractility on arterial hemodynamics: A model-based investigation. Plos One 16, e0255561–14. 10.1371/journal.pone.0255561 34339454 PMC8328319

[B59] Picone D. S. Schultz M. G. Armstrong M. K. Black J. A. Bos J. W. Chen C. H. (2021). Identifying isolated systolic hypertension from upper-arm cuff blood pressure compared with invasive measurements. Hypertension 77, 632–639. 10.1161/HYPERTENSIONAHA.120.16109 33390047

[B60] Presa J. L. Saravia F. Bagi Z. Filosa J. A. (2020). Vasculo-neuronal coupling and neurovascular coupling at the neurovascular unit: Impact of hypertension. Front. Physiol. 11, 584135. 10.3389/fphys.2020.584135 33101063 PMC7546852

[B61] Qohar U. N. A. Munthe-Kaas A. Z. Nordbotten J. M. Hanson E. A. (2021). A nonlinear multi-scale model for blood circulation in a realistic vascular system. R. Soc. open Sci. 8, 201949. 10.1098/rsos.201949 34966547 PMC8633777

[B62] Rachev A. Shazly T. (2019). A structure-based constitutive model of arterial tissue considering individual natural configurations of elastin and collagen. J. Mech. Behav. Biomed. Mat. 90, 61–72. 10.1016/j.jmbbm.2018.09.047 30352323

[B63] Ramachandra A. B. Humphrey J. D. (2019). Biomechanical characterization of murine pulmonary arteries. J. Biomech. 84, 18–26. 10.1016/j.jbiomech.2018.12.012 30598195 PMC6361676

[B64] Roman M. J. Devereux R. B. Kizer J. R. Lee E. T. Galloway J. M. Ali T. (2007). Central pressure more strongly relates to vascular disease and outcome than does bronchial pressure: The strong heart study. Hypertension 50, 197–203. 10.1161/HYPERTENSIONAHA.107.089078 17485598

[B65] Safar M. E. (2008). Review: Pulse pressure, arterial stiffness and wave reflections (augmentation index) as cardiovascular risk factors in hypertension. Ther. Adv. Cardiovasc. Dis. 2, 13–24. 10.1177/1753944707086652 19124404

[B66] Schutte R. Huisman H. W. Schutte A. E. Malan N. T. (2005). Leptin is independently associated with systolic blood pressure, pulse pressure and arterial compliance in hypertensive african women with increased adiposity: The powirs study. J. Hum. Hypertens. 19, 535–541. 10.1038/sj.jhh.1001856 15759020

[B67] Seravalle G. Grassi G. (2017). Obesity and hypertension. Pharmacol. Res. 122, 1–7. 10.1016/j.phrs.2017.05.013 28532816

[B68] Shandong Institute of Technology and Northeast Electric Power University (1979). “Engineering fluid mechanics,” in Chinese (Beijing: Water Conservancy and Electric Power Press).

[B69] Sheng W. C. Zhang Q. L. Zheng Y. X. (2021). A Direct eulerian GRP scheme for a blood flow model in arteries. SIAM J. Sci. Comput. 43, A1975–A1996. 10.1137/19m1284476

[B70] Shinde S. Mukhopadhyay S. Mukhopadhyay S. (2022). Investigation of flow in an inealized curved artery: Comparative study using CFD and FSI with Newtonian and non-Newtonian fluids. J. Mech. Med. Biol. 22, 2250009. 10.1142/s0219519422500105

[B71] Staessen J. A. Fagard R. Thijs L. Celis H. Arabidze G. G. Birkenhäger W. H. (1997). Randomised double-blind comparison of placebo and active treatment for older patients with isolated systolic hypertension. Lancet 350, 757–764. 10.1016/s0140-6736(97)05381-6 9297994

[B72] Suzuki Y. Yeung A. C. Ikeno F. (2011). The representative porcine model for human cardiovascular disease. J. Biomed. Biotechnol. 2011, 1–10. 10.1155/2011/195483 PMC3022214 21253493

[B73] Thrivikraman G. Johnson S. L. Syedain Z. H. Hill R. C. Hansen K. C. Lee H. S. (2020). Biologically-engineered mechanical model of a calcified artery. Acta Biomater. 110, 164–174. 10.1016/j.actbio.2020.04.018 32305446

[B74] Tofas T. Fatouros I. G. Draganidis D. Deli C. K. Chatzinikolaou A. Tziortzis C. (2021). Effects of cardiovascular, resistance and combined exercise training on cardiovascular, performance and blood redox parameters in coronary artery disease patients: An 8-month training-detraining randomized intervention. Antioxidants 10, 409. 10.3390/antiox10030409 33803076 PMC8001546

[B75] Tomiyama H. (2020). Does arterial stiffness reflect cardiovascular risk in the very elderly? J. Clin. Hypertens. 2, 1216–1217. 10.1111/jch.13892 PMC8029676 32558247

[B76] Vasquez E. T. Barrera F. Duenas M. S. Jimenez E. Rocuts A. Perez E. R. (2019). Association between blood viscosity and cardiovascular risk factors in patients with arterial hypertension in a high altitude setting. Cureus 11, e3925. 10.7759/cureus.3925 30937231 PMC6433084

[B77] Verdecchia P. Angeli F. Mazzotta G. Garofoli M. Ramundo E. Gentile G. (2012). Day-night dip and early-morning surge in blood pressure in hypertension: Prognostic implications. Hypertension 60, 34–42. 10.1161/HYPERTENSIONAHA.112.191858 22585951

[B78] Verdecchia P. Angeli F. Reboldi G. (2017). The tale of an innocent: Intensive treatment and the J-curve in the SPRINT trial (systolic blood pressure intervention trial). Circulation 136, 2230–2232. 10.1161/circulationaha.117.030791 29203565

[B79] Virdis A. (2018). Arterial stiffness and vascular aging: From pathophysiology to treatment, with a look at the future. High. Blood Press. Cardiovasc. Prev. 25, 135–136. 10.1007/s40292-018-0253-4 29470787

[B80] Vokó Z. Bots M. L. Hofman A. Koudstaal P. J. Witteman J. C. M. Breteler M. (1999). J-shaped relation between blood pressure and stroke in treated hypertensives. Hypertension 34, 1181–1185. 10.1161/01.HYP.34.6.1181 10601115

[B81] Waclawovsky G. Boll L. F. C. Eibel B. Alegretti A. P. Spagnol F. Paoli J. D. (2021). Individuals with controlled hypertension show endothelial integrity following a bout of moderate-intensity exercise: Randomized clinical trial. Sci. Rep. 11, 8528. 10.1038/s41598-021-87990-6 33879820 PMC8058090

[B82] Wang H. Wang L. Miao F. Gong W. Huan S. Xu C. (2020). On “pump theory” and “wave theory” of cardiac function. In Chinese. Combust. Explo. Shock. 40, 0314–0386. 10.11883/bzycj-2020-0386

[B83] Wang J. G. Staessen J. A. Gong L. Liu L. (2000). Chinese trial on isolated systolic hypertension in the elderly. Arch. Intern. Med. 160, 211–220. 10.1001/archinte.160.2.211 10647760

[B84] Warnert E. A. H. Rodrigues J. C. L. Burchell A. E. Neumann S. Ratcliffe L. E. K. Manghat N. E. (2016). Is high blood pressure self-protection for the brain? Circ. Res. 119, E140–E151. 10.1161/circresaha.116.309493 27672161

[B85] Wu N. (2021). Observation of the clinical correlation between carotid artery intima-media thickness and cardiovascular risk factors in hypertensive patients under microscope images. J. Sensors 2021, 1–14. 10.1155/2021/8919824

[B86] Wu S. Wang D. Gao J. Wang X. Cao X. (2012). “Clinical hypertension,” in Chinese (Beijing: Peking University Medical Press).

[B87] Yin T. Cheng I. Zhu X. Liao S. Zhang H. Li X. (2021). The J-curve association between blood pressure and mortality in stroke survivors. Int. J. Gen. Med. 14, 5039–5049. 10.2147/IJGM.S326301 34511987 PMC8412835

[B88] Younis B. A. Berger S. A. (2004). A turbulence model for pulsatile arterial flows. J. Biomech. Eng. 126, 578–584. 10.1115/1.1798032 15648810

[B89] Yu S. Zhang Y. (2021). The association between isolated systolic or diastolic hypertension and cardiovascular risk. J. Hypertens. Los. Angel. 39, 1552–1554. 10.1097/HJH.0000000000002857 34188001

[B90] Zareh M. Katul R. Mohammadi H. (2019). Mechanics of atherosclerotic plaques: Effect of heart rate. Cardiovasc. Eng. Technol. 10, 344–353. 10.1007/s13239-019-00413-6 30949919

[B91] Zhang L. Yang K. (1996). “Fluid-structure coupling theory,” in Chinese (Beijing: Science Press).

